# Barriers to Accessing Emergency Medical Services in Accra, Ghana: Development of a Survey Instrument and Initial Application in Ghana

**DOI:** 10.9745/GHSP-D-15-00170

**Published:** 2015-12-15

**Authors:** Nee-Kofi Mould-Millman, Sarah D Rominski, Joshua Bogus, Adit A Ginde, Ahmed N Zakariah, Christiana A Boatemaah, Arthur H Yancey, Samuel Kaba Akoriyea, Thomas B Campbell

**Affiliations:** ^a^​ University of Colorado School of Medicine, Department of Emergency Medicine, Aurora, CO, USA; ^b^​ University of Michigan Medical School, Global REACH, Ann Arbor, MI, USA; ^c^​ Washington University in Saint Louis, School of Medicine, Infectious Diseases Division, Saint Louis, MO, USA; ^d^​ Ministry of Health Republic of Ghana, National Ambulance Service, Accra, Ghana; ^e^​ Emory University School of Medicine, Department of Emergency Medicine, Atlanta, GA, USA; ^f^​ Ghana Health Service, Institutional Care Division, Accra, Ghana; ^g^​ University of Colorado School of Medicine, Department of Medicine, Division of Infectious Diseases, Aurora, CO, USA

## Abstract

**Background:**

Emergency medical services (EMS) systems provide professional prehospital emergency medical care and transportation to help improve outcomes from emergency conditions. Ghana’s national ambulance service has relatively low public utilization in comparison with the large burden of acute disease.

**Methods:**

A survey instrument was developed using Pechansky and Thomas’s model of access covering 5 dimensions of availability, accessibility, accommodation, affordability, and acceptability. The instrument was used in a cross-sectional survey in 2013 in Accra, Ghana; eligible participants were those 18 years and older who spoke English, French, or Twi. Although the analysis was mainly descriptive, logistic regression was used to identify factors associated with reported intention to call for an ambulance in the case of a medical emergency.

**Results:**

468 participants completed surveys, with a response rate of 78.4%. Few (4.5%) respondents had ever used an ambulance in prior emergency situations. A substantial proportion (43.8%) knew about the public access medical emergency telephone number, but of those only 37.1% knew it was a toll-free call. Most (54.7%) respondents believed EMTs offered high-quality care, but 78.0% believed taxis were faster than ambulances and 69.2% thought the number of ambulances in Accra insufficient. Many (23.4%) thought using ambulances to transport corpses would be appropriate. In two hypothetical emergency scenarios, respondents most commonly reported taxis as the preferred transportation (63.6% if a family member were burned in a house fire, 64.7% if a pedestrian were struck by a vehicle). About 1 in 5 respondents said they would call an ambulance in either scenario (20.7% if a family member were burned in a house fire, 23.3% if a pedestrian were struck by a vehicle) while 15.5% and 10.2%, respectively, would use any available vehicle. Those aged 18–35 years were more likely than older respondents to prefer an ambulance (odds ratio [OR], 2.27; confidence interval [CI], 1.47 to 3.68), as were those with prior ambulance experience (OR, 1.75; CI, 0.98 to 3.09) (compared with those with no prior experience) and those who believed ambulances were safer than taxis (OR, 2.17; CI, 1.1 to 4.2) (compared with those who did not hold such beliefs).

**Conclusions:**

Perceptions of public ambulance services in Accra, Ghana, are generally favorable, although use is low. Public health education to improve awareness of the toll-free medical emergency number and about appropriate use of ambulances while simultaneously improving the capacity of ambulance agencies to receive increased caseload could improve use of the EMS system.

## Abstract (precis)

Most respondents thought the number of ambulances insufficient and said they would rather use a taxi—perceived to be faster—in a medical emergency. Nevertheless, people generally had favorable attitudes of existing public ambulance services, although few knew of the toll-free emergency number and many thought it appropriate to use ambulances to transport corpses. Targeted public education, along with improved capacity of ambulance agencies to handle increased caseload, could improve use.

## BACKGROUND

Emergency medical services (EMS) are a community’s gateway to acute and emergency medical care for members of the public facing time-sensitive, condition-critical illness and injury.^1^^,^^2^ When implemented appropriately, EMS systems are an effective, frontline, public health intervention to reduce the disproportionately high morbidity and mortality in low- and middle-income countries.^1^^,^^3^^,^^4^ The formation of locally appropriate EMS systems in low-resource settings, to provide emergency care and transport, has been promoted by international bodies, such as the World Health Organization and the African Federation for Emergency Medicine.^1^^,^^2^^,^^5^^,^^6^

Thus, EMS systems, regardless of their state of development, play a critical role in the continuum of ensuing medical care.^1^^,^^2^^,^^7^ Emergency care usually begins in the community, when someone identifies a perceived emergency condition and attempts activation of the local EMS system. This ideally triggers a cascade of events resulting in a timely response of expertise, resources, and service directed to patient stabilization and/or safe emergency patient transportation to the nearest appropriate facility.^1^^,^^2^^,^^5^^,^^8^^,^^9^ The current norm in many low- and middle-income countries is, however ironic, to use a private vehicle or a taxi to transport the injured or ill person to the hospital, even when EMS has an active presence in these communities.^10^^,^^11^

Use of a private vehicle or taxi for transportation during an emergency is the current norm in many countries.

Across Africa, in-hospital and prehospital emergency care systems are being developed to serve diverse, multicultural, and multilingual populations of varying socioeconomic strata.^2^^,^^6^^,^^12^^,^^13^ For example, innovative programs in which motorbikes equipped with stretchers are activated in Malawi to transport emergency obstetric patients, and Ghana’s National Ambulance Service (NAS) provides professional crews and time-sensitive emergency transportation for patients.^9^^,^^14^^,^^15^

Ghana boasts one of sub-Saharan Africa’s thriving EMS systems. Formed in 2004, the NAS is comprised of a fully operational ambulance fleet with 160 basic life support-equipped ambulances and more than 1,200 emergency medical technicians (EMTs). It has a nationwide operational footprint, providing free emergency services to the citizens of Ghana. Within each of Ghana’s 10 regional capitals in 2013, NAS had at least 1 ambulance station and several ambulances staffed by a crew of trained EMTs. Accra, the national capital, had the largest NAS complement of ambulances and personnel, with 8 ambulances and approximately 100 EMTs.^16^^,^^17^ Still, the Accra ambulance-to-population ratio is approximately 1:250,000—a ratio that is 5–10 times below expert-recommended ratios for lower-income countries—and the mean response time is about 18 minutes.^16^^,^^17^ Injuries are the most common reason for public utilization of NAS services.^16^^,^^17^ Despite sustained growth of NAS over the past decade, annual reports have indicated low public utilization, which may be a contributor to continued poor outcomes of acute care. The reasons behind low utilization have not been studied sufficiently.^9^^,^^16^^,^^17^

This study was undertaken to characterize and quantify the range of barriers—from the demographic and psychosocial to the financial, knowledge, and cultural barriers—that prevent Ghanaian citizens in Accra from appropriately accessing local EMS resources. In Accra, we hypothesized low public opinion of ambulance services, poor knowledge of ambulance access, and thus a strong public preference to use non-ambulance means of transport during medical emergencies.

## METHODS

### Conceptual Approach

Pechansky and Thomas provide a conceptual framework of 5 dimensions that affect patient access to or entry into health care systems: availability, accessibility, accommodation, affordability, and acceptability (Box).^18^ Applications of this framework have elucidated causes for increased mortality pertaining to accessing emergency care, for emergency conditions such as sepsis and respiratory and obstetric emergencies.^19^^–^^21^

**BOX. Five Dimensions of Access to Health Care**

1. **Availability:** the relationship of the volume and type of existing services (and resources) to the clients’ volume and needs.
2. **Accessibility:** the relationship between the location of supply of services (or resources) and the location of clients.
3. **Accommodation:** the manner in which the services (or resources) are organized to meet the needs of clients and clients’ perceptions of the appropriateness of the way services are organized.
4. **Affordability:** the relationship between the cost and perceived value of services and the clients’ ability to pay.
5. **Acceptability:** the relationship of the clients’ perceptions and attitudes toward the service (or resources) to the actual characteristics of the service, as well as to the perceptions and attitudes of providers toward certain clients.

Source: Penchansky and Thomas.^18^

The domain of content and relevant elements for our survey instrument were identified by a multidisciplinary research team, including experts in emergency care, EMS, African public health, and survey design, following discussions and a review of the literature. Desired attributes of the survey instrument included comprehensiveness, simplicity in execution, standardized oral administration, and ease of comprehension by respondents, independent of their sociopolitical, cultural, and geographic background. The survey was designed to be delivered on a tablet computer (Nexus 7, Google Inc., USA) using survey software (droidSurvey 2013, Google Inc., USA) to facilitate data collection. The research team developed survey questions for each element. Most of these questions were structured to enable quantitative categorization of responses. Due to the exploratory nature of this research, open-ended questions were also included to promote less-restricted responses and provide a richer explanation of issues raised by respondents.

### Development and Validation of Survey Instrument

The research team defined the domain of the survey as all citizen-perceived barriers potentially impeding EMS access in low-resource sub-Saharan African settings. Relevant elements to the domain were screened in the context of Pechansky and Thomas’s domains of access (i.e., availability, accessibility, accommodation, affordability, and acceptability).

The survey instrument was written in English and designed to be orally administered to respondents by trained bilingual research assistants in English (the official language), French, or Twi (the most dominant local language). Each research assistant received more than 20 hours of training on the sampling technique and survey instrument, which included completing 15 supervised surveys each. The survey contained 8 main sections:

1. Respondent demographics
2. Prior experience(s) with EMS
3. Knowledge of ambulance function
4. Perceptions of ambulance performance
5. Hypothetical scenarios
6. Barriers to access
7. EMS educational intervention (in which we provided participants information about EMS)
8. Survey administration/logistics (e.g., interviewer name, location of survey, and language)

Of the 114 total questions, respondents were asked a maximum of 104, while 10 questions pertained to survey administration. The instrument had skip logic successfully embedded within it.

Early versions of the instrument underwent multiple rounds of revisions and sequential stages of validation. Three external survey experts provided feedback on its structure, including the syntax, grammar, and meaning of the chosen questions. Cognitive testing of a preliminary version of the instrument was performed in the United States (in Atlanta, Georgia, and in Ann Arbor, Michigan) and then in Ghana (in Accra and Kumasi) among 19 community members in total. During cognitive testing, feedback was provided on the meaning and clarity of questions, brevity, and ease of understanding and of responding to the survey. Pilot testing on the tablet computer was also conducted in Ghana, between April and June 2013, among 30 community members to ensure trouble-free data collection, to assess survey duration, and to resolve any impediments to conducting the survey.

According to the 49 community members and the 6 expert reviewers who participated in both phases of pilot testing and qualitative critique of the survey, the survey instrument passed face validity and content validity, and it was deemed sound with regards to construct validity and translation. To test the instrument’s consistency when administered by different surveyors, the instrument was tested by multiple surveyors over 2 days: 2 survey administrators on day 1, and 3 survey administrators on day 2. Interrater reliability measurements of key variables yielded Krippendorff’s alpha scores between 0.66 to 1, suggesting good to excellent interrater reliability.^22^^,^^23^ Intrarater and test-retest reliability were non-applicable because the EMS educational intervention (an integral part of the survey mission) would skew respondents’ subsequent answers and introduce error into tests of reliability. All initially stated desired attributes were satisfied by the final design of the instrument.

### Sampling

Surveys were conducted in a representative cross-section of the general public in Ghana’s capital city, Accra, from June to August 2013. Accra is a coastal city in southern Ghana, and the metropolitan area had a population of approximately 1.8 million in 2012, distributed over 173 square kilometers.^24^^,^^25^

The rationale for selecting Accra was that the Accra metropolitan area has the highest concentration of NAS ambulances and the highest population volume and density in Ghana.^26^^,^^27^ Therefore, Accra qualified as a high-yield survey study site in which to reach an initial understanding of barriers to EMS access in the urban Ghanaian population.

To conduct balanced community-based random sampling, we replicated the population survey technique used in the 2003 and 2008 versions of the Ghana Demographic and Health Survey.^28^ In this approach, the Accra metropolitan area was divided into 11 geographic clusters, termed enumeration areas (Figure). To ensure valid statistical analysis could be conducted within each enumeration area, a target sample size of approximately 40 in each of the 11 areas was chosen, giving an overall sampling size of 440.

**FIGURE.**
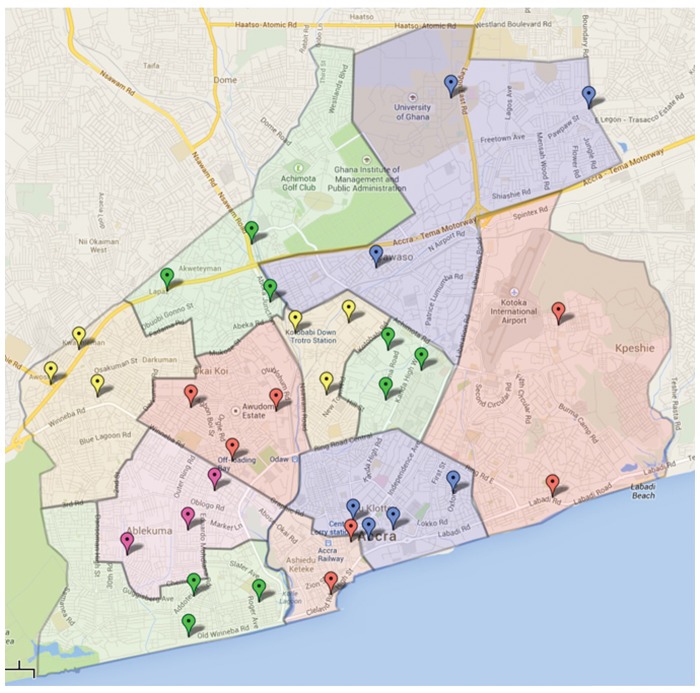
Survey Locations Within the 11 Demographic and Health Survey Enumeration Areas for Accra, Ghana

Two research assistants (JB, CA) were trained by the study’s lead investigators. The research assistants were fluent in English (both JB and CA), French (JB), and Twi (CA). In order to attain as broad a sample as possible, diverse areas including the road side, commercial areas, residential areas, schools, places of worship, and recreational areas were used as recruitment sites. By sampling every fifth person within each recruitment site and by using a goal of 6 to 8 interviews per site, an approximately equal number of people were interviewed within each enumeration area.

### Inclusion and Exclusion Criteria

All persons 18 years of age and older, located within the 11 enumeration areas during our survey period, were eligible to participate after providing informed consent. Respondents who did not speak English, French, or Twi were excluded.

### Data and Statistical Analysis

After we conducted the survey, we exported the data from the droidSurvey database into Microsoft Excel (2007, Redmond, WA). Quantitative data from closed-ended questions were cleaned and coded. Qualitative data from open-ended questions were reviewed by the authors and coded individually, into themes. Following initial coding, 2 authors (NM and SR) compared codes, discussed and resolved discrepancies, and categorized the codes into thematic areas. Missing data were noted as such, and no imputation was performed for missing variables. Cleaned and coded data were exported into a statistical software program, Stata (Stata Corp, College Station, TX), for analysis. Descriptive statistics were performed for quantitative data, using frequencies, means, and standard deviations (SDs), as appropriate.

In the survey, participants were presented with 2 hypothetical emergency situations and asked how they would prefer transport of the victim to the hospital. In the first scenario, the participant witnessed a pedestrian on the street struck by a car, implying accessibility to many private and commercial vehicles. In the second scenario, a relative was burned badly in a house fire, implying limited access to private or commercial vehicles. The primary outcome for regression analysis was the response to the question, “If you saw a pedestrian hit by a car and they needed to go to a hospital immediately, how would you get them to the hospital?” Answers were categorized, then dichotomized to “ambulance” (combining the NAS ambulance and other ambulance options) and “non-ambulance,” then were analyzed using logistic regression.

An analytic model was developed to assess the effect of independent variables on this primary outcome variable. Independent variables in our model included respondent’s age, sex, education level, prior experience with ambulances, knowledge of local ambulance services, knowledge of appropriate ambulance functions, and knowledge of the local emergency access number, all of which were assessed as part of the survey. To explore the relationship between independent variables and the outcome variable (decision to call an ambulance), descriptive cross tabs with chi-square analysis were performed. Variables were selected according to previous literature and the expert opinion of the authors. Variables that were found to be significant in bivariate analysis at the 0.1 level were entered into a multivariate model.

### Ethical Approval

Ethical approval was obtained from the following human subjects committees: Colorado Multiple Institutional Review Board, University of Michigan Institutional Review Board, and the Ghana Health Service Ethical Review Committee.

## RESULTS

Of 597 people who were approached, 468 completed the survey, yielding a survey response rate of 78.4%. Almost all (>90%) persons who refused participation did not give a reason for why they declined to consent. Respondents were sampled from all 11 enumeration areas, with between 38 and 57 respondents in each area. The majority of the respondents were surveyed in business locations (n = 204, 43.6%), followed by the street (n = 135, 28.8%) and residential locations (n = 89, 19.0%). The remainder (n = 33, 7.1%) were surveyed in locations coded as “other,” mainly postgraduate schools, such as law school. Location data were missing for 7 participants (0.85%). Most of the surveys were conducted in English (n = 273, 58.3%), but a substantial proportion were translated (n = 195, 40.2%), virtually all in Twi (n = 184 in Twi, n = 1 in French, n = 10 not documented).

### Background Demographic Characteristics

Slightly more than half of the participants who completed the survey were men (n = 248, 53.0%) (Table 1). The mean age was 34.9 years (SD, 12.9), with the majority (n = 277, 59.2%) of respondents between 18–35 years. The large majority (n = 448, 95.7%) resided in the Accra metropolitan area. Only 53 participants (11.3%) owned a car, but 453 (96.8%) owned a cell phone. About one-third (n = 159, 34.0%) of the respondents had attained post-secondary education.

**TABLE 1:** Demographic Characteristics of Survey Respondents, Accra, Ghana (N = 468)

	No. (%)		No. (%)
**Sex**		Ewe	85 (18.1)
Male	248 (53.0)	Hausa	73 (15.6)
Female	220 (47.0)	Other	145 (31.0)
**Age, y**		**Education**	
18–35	277 (59.2)	None	32 (6.8)
36–50	128 (27.3)	Primary	108 (23.1)
51+	63 (13.5)	Secondary	167 (35.7)
**Residence**		Technical	19 (4.1)
Accra metropolitan area	448 (95.7)	Certificate	66 (14.1)
Other	20 (4.3)	Diploma	26 (5.6)
**Cell phone ownership**		University	42 (9.0)
Yes	453 (96.8)	Post-University	6 (1.3)
No	15 (3.2)	Missing	2 (0.4)
**Car ownership**		**Occupation**	
Yes	53 (11.3)	Service and sales worker	231 (49.4)
No	415 (88.7)	Student	83 (17.7)
**Languages spoken**^a^		Craft and related trade	54 (11.5)
Twi	413 (88.2)	Technicians and associated profession	37 (7.9)
English	391 (83.5)	Industrial and machinery operator	15 (3.2)
Ga	220 (47.0)	Other	48 (10.3)

a Percentages do not total to 100%, given multilingual respondents.

### Prior Ambulance Use

In the preceding 5 years, 350 (74.8%) respondents had had a personal experience and/or had witnessed an experience with either or both a medical (n = 246, 57.7%) or traumatic (n = 205, 47.1%) incident that required acute or emergency care. Of those prior incidents requiring acute or emergency care, 402 (89.1%) resulted in transportation to the hospital. Only 18 (4.5%) respondents had ever used an ambulance in the emergency situation (Table 2). The rest of respondents had used taxis (n = 195, 48.5%), private cars (n = 49, 12.2%), or other (n = 112, 27.9%) forms of transportation. Of the 18 respondents who recalled using an ambulance, 4 (22.2%) had called the public access emergency medical number and the rest (n = 14, 77.8%) had used alternative means to access the service (data not shown). In total, 69 (14.7%) respondents recalled being inside an ambulance in the past 5 years.

While 75% of respondents had had a prior experience with an emergency incident, only 5% had ever used an ambulance.

**TABLE 2:** Prior Experiences in Past 5 Years With Transportation During an Emergency Among Survey Respondents (N = 350 Respondents for a Total of 402 Incidents)^a^

	Type of Emergency		
	Injury, No. (%)	Illness, No. (%)	Total, No. (%)
**Type of transportation to hospital used**			
Ambulance	15 (9.6)	3 (1.2)	18 (4.5)
Taxi	85 (54.5)	110 (44.7)	195 (48.5)
Private car	22 (14.1)	27 (11.0)	49 (12.2)
Tro-Tro (minibus)	8 (5.1)	60 (24.4)	68 (16.9)
Motorbike	4 (2.6)	0 (0.0)	4 (1.0)
Walked	2 (1.3)	38 (15.4)	40 (10.0)
Don’t remember	6 (3.8)	7 (2.8)	13 (3.2)
Missing	14 (9.0)	1 (0.4)	15 (3.7)
**TOTAL**	**156 (38.8)**	**246 (61.2)**	**402 (100)**
**How transportation was accessed**			
Waved down taxi	5 (4.1)	0 (0.0)	5 (1.5)
Someone waved taxi	14 (11.5)	0 (0.0)	14 (4.3)
Walked to ambulance station	1 (0.8)	0 (0.0)	1 (0.3)
Used own car/family drove	2 (1.6)	0 (0.0)	2 (0.6)
Shouted/yelled for help	19 (15.6)	0 (0.0)	19 (5.8)
Called known contact	4 (3.3)	30 (14.5)	34 (10.3)
Sent someone to find transport	42 (34.4)	54 (26.1)	96 (29.2)
Went myself to find transport	9 (7.4)	114 (55.1)	123 (37.4)
Health care provider called transport for me	0 (0.0)	2 (1.0)	2 (0.6)
Don’t remember	11 (9.0)	6 (2.9)	17 (5.2)
Missing	15 (12.3)	1 (0.5)	16 (4.9)
**TOTAL**	**122 (37.1)**	**207 (62.9)**	**329 (100)**

a Respondents could have experienced either a traumatic (injury) or medical (illness) emergency or both.

### Knowledge and Perceptions of Ambulance Services

Table 3 details respondents’ knowledge and perceptions of ambulance services in Ghana, according to the 5 dimensions of access defined by Pechansky and Thomas.

**TABLE 3:** Knowledge and Perceptions of Ambulance Services Among Survey Respondents (N = 468)

	No. (%)		No. (%)
**Availability**		**Affordability**	
Knew at least one ambulance company	425 (90.8)	Respondents who knew of the public emergency number (n = 205) and thought the cost of calling that number was:	
Knew about government ambulance service	213 (45.5)	Free (i.e., toll free)	76 (37.1)
Able to name the National Ambulance Service (NAS)	26 (5.6)	Same as a regular call	31 (15.1)
Thought the number of ambulances in Accra insufficient	324 (69.2)	Less expensive than a regular call	39 (19.0)
**Accessibility**		More expensive than a regular call	12 (5.9)
Knew about public emergency access number	205 (43.8)	Did not know the cost	47 (22.9)
Knew public access number is 1-9-3	16 (3.4)	More likely to call 1-9-3 in an emergency if the call was free	167 (35.7)
Expected NAS ambulance response time during peak traffic hours		Thought the cost of government ambulance service was:	
≤15 minutes	165 (35.3)	Free	14 (3.0)
Between 16 and 59 minutes	271 (57.9)	Affordable	235 (50.2)
≥60 minutes	32 (6.8)	Too expensive	10 (2.1)
Expected NAS ambulance response time during non-peak traffic hours		Thought the cost of private ambulance service was:	
≤15 minutes	311 (66.5)	Cheap	6 (1.3)
Between 16 and 59 minutes	154 (32.9)	Affordable/reasonable	16 (3.4)
≥60 minutes	3 (0.6)	Too expensive	235 (50.2)
**Accommodation** a		Did not know	206 (44.0)
Identified at least one appropriate indication (as defined by NAS) for accessing an ambulance	444 (96.3)	**Acceptability**	
Thought use of ambulance to transport corpses would be appropriate	108 (23.4)	Believed ambulance technicians in Accra offered high-quality care	256 (54.7)
Thought ambulances in Accra were currently being used to:		Believed it is safer to go to the hospital by ambulance than by taxi in Accra	378 (80.8)
Transport persons with medical illnesses	223 (48.4)	Believed it is faster to go to the hospital by taxi than by ambulance in Accra	365 (78.0)
Transport injured people	161 (34.9)	Believed it is “better” to go to the hospital by ambulance than by taxi in Accra	403 (86.1)
Conduct interfacility transfers	90 (19.5)		

a N = 461 as there were 7 missing tresponses.

#### Availability

213 (45.5%) respondents knew specifically about the government ambulance service, but only 26 (5.6%) correctly knew it was called the National Ambulance Service. The majority (n = 324, 69.2%) thought there was an insufficient number of ambulances in Accra.

69% of respondents thought the number of ambulances in Accra insufficient.

#### Accessibility

205 (43.8%) knew the existence of a public access medical emergency telephone number (1-9-3). During *peak traffic hours*, 165 (35.3%) of the respondents indicated that it would take a government ambulance (NAS) 15 minutes or less to arrive at the location of their incident, while 32 (6.8%) stated it would take 60 minutes or more.

#### Accommodation

To gauge participants’ perceptions of the client needs that ambulance services were currently organized to meet, we asked participants what they perceived ambulance services were currently used for in Ghana. Eight general themes emerged, centered around providing care and transportation to: (1) people with medical emergencies, (2) people with injuries, (3) burn victims, (4) obstetric patients in labor or with complications, (5) those with other emergency conditions, (6) patients requiring interfacility transportation, (7) provide general prehospital emergency care to the population, and (8) provide other specialized services. Participants most commonly reported that ambulances were currently used as transportation for medical illnesses (n = 223, 48.4%), for injuries (n = 161, 34.9%), and for interfacility transfers (n = 90, 19.5%). A substantial number (n = 108, 23.4%) thought ambulances should be used as hearses to transport dead bodies (but NAS policies do not permit or accommodate this). Selected responses from the open-ended question, “What are ambulances currently used for in Ghana?”, are listed in Table 4.

**TABLE 4:** Selected Responses About Current Use of Ambulances in Ghana According to Appropriateness of the Use

Appropriate Use of Ambulance	Inappropriate Use of Ambulance
“Because it is an emergency the ambulance is necessary since it will get to hospital faster than taxi.”	“I think the ambulance is to be for the dead … the sick and the injured should have their own special car.”
“Ambulances should be used to help those in need of health care, because we do not have enough hospitals and the ambulances can provide first aid.”	“For the corpse you can only use the ambulance, the police will arrest you if you take a dead body in another vehicle.”
“That is the job of the ambulance to save life because they have first aid in the ambulance to help you before getting to hospital.”	“For the pregnant women in labor it is better to go by taxi to get to the hospital quickly”
“Ambulances should be used to pick up the sick in the communities, but the government does not have enough so we use taxis.”	“It is not nice to put a corpse in a regular car.”
“The ambulance is supposed to be there for emergencies at home, schools, and everywhere.”	“That is what I have seen the ambulances do. I fear the dead so I could not be in the same car that has carried the dead so corpses should be carried in an ambulance.”
“Because there are not enough ambulances we only use the ambulance for emergencies, but if there were enough then we should use ambulances for critical illness.”	“The ambulance should be able to convey even those with mild sickness …”

#### Affordability

Of those respondents who knew the existence of a public-access number for medical emergencies, only 76 (37.1%) knew it was a toll-free call. About one-third (n = 167, 35.7%) of all the respondents indicated they would be more likely to call the 1-9-3 number in an emergency if they knew the call was toll free. When asked about their perceived cost of ambulances as a potential barrier, the majority of subjects indicated that government ambulances were free or affordable (n = 249, 53.2%) and that private ambulances were too expensive (n = 235, 50.2%).

Of those respondents who knew about the public access emergency number, only 37% knew it was toll free.

#### Acceptability

The majority (n = 256, 54.7%) believed the EMTs offer high-quality care, and most (n = 403, 86.1%) believed it is overall “better” to go by ambulance in an emergency. However, the majority also thought taxis are faster than ambulances in Accra (n = 365, 78.0%).

### Hypothetical Scenarios

Of the 459 total responses to both hypothetical questions, 57 (12.4%) respondents reported they would call for an ambulance (either NAS or other) in both hypothetical scenarios (witnessing a pedestrian severely struck by a vehicle and a family member being burned badly in a house fire) (Table 5). The most common response was to use a taxi in both scenarios (n = 225, 49.0%) while 21 (4.6%) stated they would take any available vehicle as a means of emergency transportation in both events.

In hypothetical emergency scenarios, respondents most commonly reported taxis as the preferred transportation mode.

**TABLE 5:** Transportation Preferences in Hypothetical Emergency Scenarios: Concordance in Survey Responses to Both Scenarios (N = 459 Responses)

Preferred Transportation if Pedestrian Struck, No. (%)	Preferred Transportation if Family Member Burned, No. (%)					
	NAS Ambulance	Other Ambulance	Taxi	Any Available Vehicle	Tro-Tro (minibus)	Total
NAS ambulance	5 (1.1)	1 (0.2)	4 (0.9)	2 (0.4)	0 (0.0)	**12 (2.6)**
Other ambulance	18 (3.9)	33 (7.2)	37 (8.1)	7 (1.5)	0 (0.0)	**95 (20.7)**
Taxi	13 (2.8)	17 (3.7)	225 (49.0)	41 (8.9)	1 (0.2)	**297 (64.7)**
Any available vehicle	0 (0.0)	7 (1.5)	19 (4.1)	21 (4.6)	0 (0.0)	**47 (10.2)**
The car that hit the pedestrian	0 (0.0)	1 (0.2)	7 (1.5)	0 (0.0)	0 (0.0)	**8 (1.7)**
**Total**	**36 (7.8)**	**59 (12.9)**	**292 (63.6)**	**71 (15.5)**	**1 (0.2)**	**459 (100.0)**

Abbreviation: NAS, National Ambulance Service.

An analytic model was developed in which the likelihood of calling an ambulance after witnessing a pedestrian-auto collision (outcome variable) was assessed as a function of respondent demographics of age, sex, education level, cell phone ownership, car ownership, prior ambulance experience, knowledge of ambulance function, knowledge of cost of the 1-9-3 call, and perception of ambulances (safe, fast, better). Age of respondent (in years) was found to be negatively associated with the likelihood of calling for an ambulance (Table 6). This response was then categorized into 3 age variables: 18–35, 36–50, and over 51 years. Being between the ages of 18 and 35 (*P* = .01) and believing ambulances were safe means of emergency transport (*P* = .02) were significantly associated with reported likelihood of respondents to call for an ambulance in the case of an emergency. Prior experience with an ambulance (*P* = .06) was positively associated with likelihood of calling for an ambulance, but the association was not statistically significant. Those respondents aged 18–35 were 2.3 times as likely to call for an ambulance (odds ratio [OR], 2.27) as older respondents, and those who believed an ambulance is safer than a taxi were over 2 times as likely to report they would call for an ambulance (OR, 2.17) as those who did not hold such beliefs, while those who had personal experience with an ambulance were 75% more likely to report they would call for an ambulance than those with no prior experience (OR, 1.75). No other statistically significant association was discovered between any other variable and likelihood of calling an ambulance in the pedestrian injury scenario.

**TABLE 6:** Likelihood of Calling an Ambulance in Hypothetical Pedestrian-Auto Collision: Results of Logistic Regression Analysis

	OR (95% CI)	*P* Value
Personal experience with ambulance	1.75 (0.98, 3.09)	.06
Aged 18–35	2.28 (1.47, 3.68)	.001
Believe ambulance safer than taxi	2.17 (1.12, 4.19)	.02

Abbreviations: CI, confidence interval; OR, odds ratio.

A similar regression was repeated using the second hypothetical scenario (in which a family member was burned in a house fire), with the respondents’ answers to how they would transport that patient to the hospital as the outcome variable, while maintaining the original independent variables (listed above). Results were similar (data not presented).

## DISCUSSION

Although the significant burden of acute disease in Ghana suggests a large need for ambulance services, calls for ambulances from the public are disproportionately lower.^9^^,^^15^^,^^16^^,^^29^ Our study has elucidated contributing factors to this issue in Accra, where study participants most commonly indicated they would call a taxi—perceived to be faster than ambulances—in hypothetical traumatic emergency situations. The majority of participants thought there was an insufficient number of ambulances. Yet most participants had favorable opinions of existing ambulances, indicating they provided high-quality care and that they were safer and ultimately better than taxis. Only a minority, however, knew of the toll-free public access number, and many thought that ambulances should be used to transport corpses. With awareness of proper use of ambulance services and how to access it, along with adequate numbers of ambulances per population, use of ambulance services in emergency medical situations may improve. Our main findings, interpretations, and their significance are discussed below in the context of respondents’ prior experiences, preferences, and perceived barriers and facilitators.

### Prior Emergency Medical Experiences and Use of Ambulance Services

Up to 75% of respondents reported experiencing a medical or traumatic emergency within the preceding 5 years. Among these, only about 5% had used an ambulance for emergency transportation to a health care facility. This is a lower proportion of ambulance use than was found in prior studies in Ghana that indicated ambulance use by the general population in 8% to 12% of emergencies.^9^^,^^11^ This difference may be accounted for by respondent recall bias, although we do acknowledge that emergency situations are often major life events and less likely forgotten; however, we have no way to assess to what extent participants were accurately recalling events. In addition, this study excluded cases of inter-hospital ambulance transfers, which are relatively common in Ghana and were included in prior studies.^9^^,^^11^^,^^16^ Nonetheless, the results confirm a generally low rate of ambulance access requests and utilization for medical and traumatic emergencies in a resource-constrained African setting.

### Preferences for Modes of Emergency Transportation

When presented with the study’s hypothetical emergency injury scenarios (pedestrian hit by a car and person burned in a house fire), relatively few (12%) Ghanaians stated they would call for an ambulance in both scenarios, while 49% said they would rather use a taxi in both scenarios. These findings may be explained by the fact that 75% of respondents perceived taxis to be a “faster” form of prehospital transport than ambulances. Although there is no prior research assessing taxi response times in Ghana, given the ubiquitous presence of taxis and relative paucity of ambulances in 2013, it seems reasonable to assume a faster response time from the former.^16^ Paradoxically, respondents generally had favorable opinions of existing ambulance services in Accra: over half (55%) felt that EMTs offer “high-quality” care, 81% acknowledged that ambulances were in fact the “safer” form of emergency transport, and 86% affirmed that ambulances were ultimately the “better” way to be transported during emergencies. However, 69% of our sample felt the number of ambulances in the Accra metropolitan area insufficient. This leads to the inference that when an adequate number of ambulances exists, and citizens are made aware of this service, individuals (who appear to generally understand the appropriate indications to access an ambulance) may be inclined to actively seek access to them more often for primary, or scene, responses. Of note, given the high percentage of respondents who indicated some preference for taxis, a complementary, practical non-ambulance solution for safer emergency transport may include mass training of taxi drivers in first aid, appropriate destination hospital selection, and safe emergency transport (successfully demonstrated by Mock et al. in a small pilot study in Kumasi, Ghana, in the 1990s).^9^^,^^10^

When adequate numbers of ambulances exist and citizens are aware of the service, individuals may be more inclined to use ambulances.

### Perceived Barriers and Facilitators to Accessing Ambulances

Although 44% of respondents who knew the existence of a public access number in Accra, only 37% knew it was toll free, and 36% stated they would be more likely to call if they knew the call was free. Half believed NAS (the government ambulance service) was affordable, but very few (3%) knew it was a free service. Misperceptions about telephone access and cost of ambulance services are indicative of a general paucity of education among Ghanaians in Accra, and this is most likely generalizable to citizens across the nation. Issues within Ghana that focus on telephone-based access to emergency medical services through emergency medical dispatch are the subject of a parallel study by the investigators of this study.

We demonstrated that those with prior personal experience with an ambulance service, who were younger in age, or who believed ambulances to be a safer means of transport were more likely to report they would call for an ambulance in the event of a medical emergency.

Several previously hypothesized barriers were not perceived by our participants as obstacles to accessing EMS services, including poor knowledge of ambulance companies and appropriate indications to access ambulances. Over 90% of respondents were aware of the existence of at least one ambulance company in Accra, and about half knew specifically of NAS. Over 90% of surveyed citizens knew at least one appropriate indication for accessing an ambulance. Interestingly, 23% of respondents also inappropriately suggested that ambulances in Ghana should be used to transport corpses, a long-standing accepted practice in Ghana that was outlawed in 2012. This warrants special mention as many respondents felt ambulances were generally vehicles for transporting corpses, a common misperception that may prevent appropriate ambulance access. This deep-rooted sociocultural perspective may persist until this historic practice is completely aborted in Ghana and purposeful public information reverses the misperception.

About 1 in 5 respondents thought ambulances should transport corpses.

**Figure:**
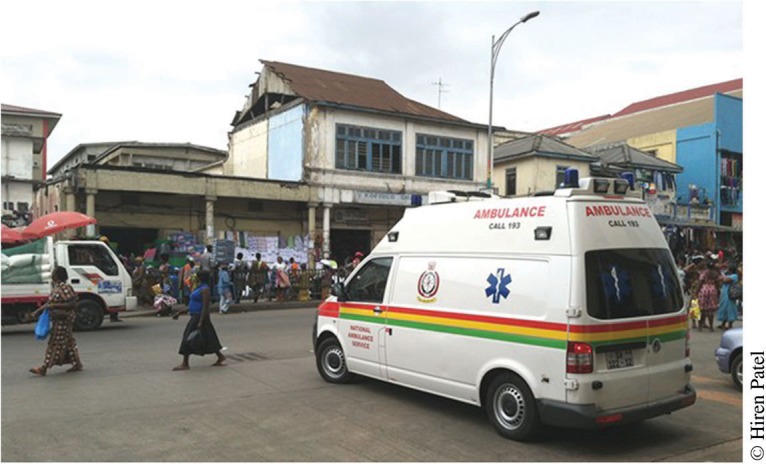
A NAS ambulance on standby at a busy marketplace in central Accra, Ghana.

Prior to this study, only one published report in the African emergency care literature was found that sought to assess barriers to accessing prehospital emergency care by a medically undifferentiated population.^30^ That study was undertaken in Libreville, Gabon, in 2009, where investigators conducted a brief 9-question oral interview of a small (N = 25) convenience sample of patients and visitors at a local emergency center. Qualitative results from this study indicated that misperceptions, lack of awareness, alternative forms of transport, and cost were all barriers to accessing prehospital resources. While our results support many of these findings, cost does not appear to be as substantial a barrier as in this previous study. Further, the majority of our study’s population had high awareness of the service and confidence in the service’s care and safety. However, the specifics about how to access the service, indicated by the low prevalence of awareness of the free public access phone number, reflect a population-based problem of access to care in Ghana.

### Possible Interventions to Improve Use of Ambulance Services

If the culture of seeking formal emergency transportation and professional prehospital medical care does not shift among populations, EMS systems risk failing in their mission to save lives when time-sensitive expertise-driven care is required. Fortunately, our findings bear favorable implications for public health education as a means to appropriately improve access to and use of ambulance services in Ghana, given the preponderance of short-term modifiable factors. For example, focused public education about the toll-free number, ambulance safety, and response or transport times, delivered via media consumed by persons aged 18–35, may be one high-yield targeted method to improve appropriate public ambulance utilization, once adequate ambulance response units exist in the near future. This may be accomplished as part of public media campaigns and/or incorporated into school-based education. However, there has purposefully been no large-scale public educational efforts by the Ghana Ministry of Health or NAS given the small capacity of this ambulance service relative to its large jurisdictional responsibility.

It is noteworthy that while the development of an EMS system is important and prehospital care confers a survival benefit, there must be concurrent improvement in in-hospital emergency care to synergistically improve patient outcomes. Emergency medicine is a recognized medical specialty in Ghana, and while specialist training has been ongoing in Kumasi, it is yet to commence in Accra where there is a paucity of emergency medicine specialist physicians.^9^^,^^15^

### Adaptation of the Survey Instrument

While this survey instrument was designed for application in Ghana, plans are underway to adapt it to other African settings in which EMS leaders, administrators, and researchers desire to scientifically understand and quantify barriers to access of their local emergency medical services in order to increase the public’s appropriate use of EMS services.

### Limitations

We do note heavier sampling in business and academic centers, which may have introduced a selection bias toward more-educated individuals, thereby making our sample less representative of the Accra population. Analysis of the Accra study population’s demographics indicate a high similarity to the general Ghanaian population; however, our conclusions are less generalizable to other urban areas in Ghana and least generalizable to non-urban areas in Ghana. Further, recall bias and decisions based on hypothetical emergency situations do not necessarily correlate with actual practice, and this is an unavoidable shortcoming in a survey.

## CONCLUSIONS

Across Africa, EMS systems are developing rapidly in an effort to address the large burden of growing acute injury and endemic disease. This article describes the first known successful development and application of a robust community-based survey instrument to quantify the demographic, perceptual, and experiential factors that may prevent an African subpopulation from accessing critical EMS resources. Findings from this novel study indicate generally favorable perceptions of public ambulance services in Accra. Once ambulance agencies are operationally poised to receive an increased call volume and caseload, public education should be targeted to address selected topics, for example, to improve awareness of the toll-free public access number and about the inappropriateness of transporting corpses, thereby positively influencing citizens’ decisions to call for an ambulance. The present findings also support consieration of reconfiguring the public ambulance service to encompass trained taxi services as first-responders, which has been previously pilot tested in Ghana with operational and educational success.

Future work will include deploying the survey in rural Ghanaian settings and other African countries developing EMS systems.

## Notes

**Peer Reviewed**
